# Comparison of Intraoperative Outcomes between Single-incision Robotic Cholecystectomy and Multi-incision Robotic Cholecystectomy

**DOI:** 10.7759/cureus.5386

**Published:** 2019-08-13

**Authors:** Philip Schertz, Subhasis Misra, David Livert, Joanne Mulligan, Chand Rohatgi

**Affiliations:** 1 Surgery, Brandon Regional Hospital, Brandon, USA; 2 Surgery, Easton Hospital, Easton, USA

**Keywords:** robotic surgery, minimally invasive, robotic cholecystectomy

## Abstract

Background

The aim of this study was to evaluate the differences in the key surgical factors for single-incision robotic cholecystectomy (SIRC) and multi-incision robotic cholecystectomy (MIRC).

Methods

A retrospective data review from August 2013 to April 2018 consisting of 104 SIRC and 105 MIRC cases was done considering factors including patient gender, age, operating time (skin incision to skin closure), robotic console time (docking to undocking), the preoperative diagnosis for surgery, any complications in surgery, length of stay (LOS), and estimated blood loss (EBL). Procedures with conversion away from original robotic cholecystectomy approach were excluded. Chi-square analysis (*p-*value: 0.05) was run between the two data sets.

Results

A total of 209 robotic cholecystectomy cases were reviewed since 2013. We found significantly less time with single-incision compared to multi-incision (single incision = 94.0 minutes, multi-incision = 99.9 minutes, *p *= 0.016) and EBL (single-incision = 11.52 mL, multi-incision = 17.17 mL, *p *= 0.004). There was no significant difference in age or robotic console time. The most common indication was symptomatic cholelithiasis overall, with equal cases of dyskinesia in single-incision approach, although there was no significant difference in indication between the two approaches. Intraoperatively, there was marginally significant use of irrigation in multi-incision (multi-incision 45 [42.9%], single-incision 31 [29.8%], *p *= 0.0499) and no difference in Firefly, perforation, or intraoperative cholangiogram use. LOS results showed significant decreased stay in SIRC cases (single-incision 84 outpatients [80.8%], multi-incision 75 [71.4%]; *p *= 0.0379).

Conclusions

SIRC and MIRC are both safe and feasible ways to remove the inflamed/dysfunctional gallbladder. SIRC is associated with less operative time, less blood loss, and shorter hospital stay.

## Introduction

The da Vinci system (Intuitive Surgical, Inc., Sunnyvale, CA) has been used to perform cholecystectomies since 1997, and single-incision robotic cholecystectomy (SIRC) was introduced in 2011 to overcome the previous limitations from a multi-incision robotic cholecystectomy (MIRC) [1-2]. SIRC has been favored in cholecystectomies because of the minimal scarring visible by hiding the entry point in the umbilicus and reduction in postoperative pain [3]. As an alternative to MIRC, SIRC has foreseeable benefits, but the real clinical benefits for patients remain a matter of debate. The primary pitfalls of SIRC have been associated with incisional/trocar site hernias and visceral injuries related to the larger midline incision [2,4]. SIRC has improved cosmetic appearance although the intraoperative difficulty is higher compared to MIRC, which may not offset the benefit especially in pediatrics [5]. Given the limited research, this study attempts to compare SIRC and MIRC cases in a retrospective approach at a single-community hospital across nine surgeons.

## Materials and methods

A retrospective review of operative records of 104 SIRC and 105 MIRC cases from August 2013 until April 2018 was performed. We collected data from all patients receiving robotic cholecystectomies during this time. The same standardized surgical technique was used for all patients and all operations were performed in house by nine different surgeons. Our data collection included the following variables: patient gender, age, operating time (skin incision to skin closure), robotic console time (docking to undocking), the preoperative diagnosis for surgery, any complications in surgery, length of stay (LOS), and estimated blood loss (EBL). We compared the outcomes of the two methods based on these variables. Exclusion criteria included any patients who underwent intra-operative conversion from robotic to either laparoscopic or open surgery; these included four single-incision and six multi-incision over the time frame of the study. We were unable to obtain data prior to 2014, and the most recent cases over the last six months did not have console time recorded. A literature review prior to data collection correlated our data collection with previous comparisons of robotic surgery techniques.

Statistical analysis measured means and standard deviations. Chi-square test was done. *P-*values of <0.05 were considered to represent statistically significant differences in data. The significance was used to prove or disprove the null hypothesis that there was no difference in outcomes and indications in MIRC and SIRC. This study was approved by the Easton Hospital’s institutional Review Board.

Surgical techniques

Single-incision Robotic Cholecystectomy

The patient was brought to the operating room and positioned in the reverse Trendelenburg position with arms tucked at the sides. Nursing staff, a resident physician, and often a physician assistant trained in the robotic platform was present. A midline incision was made through the umbilicus with a single-site port placed with the target anatomy facing the camera. The robotic arms were placed over the patient with the robot resting by the patient’s right shoulder. An assistant port was inserted and used to retract the gallbladder cephalad, while the triangle of Calot was dissected. After achieving the critical view of safety, the cystic duct and cystic artery were clipped and divided. The gallbladder was dissected off the gallbladder fossa and removed with the umbilical port once the dissection was finished. If there was bile spillage, the fossa was irrigated until clear irrigant was suctioned before removing the port. The fascia defect was closed.

Multi-incision Robotic Cholecystectomy

The approach is similar to the single-incision approach with the exception that a single midline incision is not made. Instead, the peritoneum is entered through direct cutdown, video-assisted trocar insertion, or Veress needle insertion for initial pneumoperitoneum followed by trocar insertion. Peritoneum access was chosen based on surgeon preference. After the initial port placement, the remaining three to four ports were placed under direct visualization aimed at the target anatomy. Once all ports were in, the robot was docked similar to above. The gallbladder was removed through a 10-mm port. The fascia defect of the 10-mm port sites was closed.

## Results

Among the 104 SIRC cases, 78 (75%) were women and 26 (25%) were men, and among the 105 MIRC cases, 75 (71.4%) were women and 30 (28.6%) were men. The average age in SIRC was 53 years and MIRC was 52 years. There was no significance in the age selection for each approach.

We found that SIRC had a less operative time, (M = 94.0 minutes, SE = 20.9) compared to MIRC (M = 99.9 minutes, SE = 30.3), and the EBL was less with single-incision (M = 11.52 mL, SE = 9.717) vs multi-incision (M = 17.17 mL, SE = 23.686) by a significant margin (*p *= 0.016 & *p *= 0.004 respectively). Console time though was not significantly less (*p *= 0.370) for SIRC (M = 41.1 minutes, SE = 16.0) vs. MIRC (M = 44.7 minutes, SE = 9.1; Tables 1 & 2). 

**Table 1:** Single-incision robotic cholecystectomy and multi-incision robotic cholecystectomy findings SIRC, single-incision robotic cholecystectomy; MIRC, multi-incision robotic cholecystectomy

	Arm	N	Mean	Standard Deviation	Standard Error Mean
Console time	SIRC	98	41.10	15.941	1.610
	MIRC	93	44.70	19.131	1.984
Operation time	SIRC	104	94.04	20.931	2.052
	MIRC	105	99.89	30.225	2.950
Age	SIRC	104	53.16	18.350	1.799
	MIRC	105	51.99	18.330	1.789
Estimated blood loss	SIRC	104	11.52	9.717	0.957
	MIRC	105	17.17	23.686	2.311

**Table 2:** Statistical analysis of single-incision robotic cholecystectomy and multi-incision robotic cholecystectomy findings *indicates significance; SIRC, single-incision robotic cholecystectomy; MIRC, multi-incision robotic cholecystectomy

	SIRC	MIRC	P-value
Console time (min)	41.1+/-16.0	44.7+/-19.1	0.370
Operative time (min)	94.0 +/- 20.9	99.9 +/- 30.3	0.016*
Age (years)	53.16 +/- 18.35	51.99 +/- 18.33	0.644
Estimated blood loss (mL)	11.52 +/- 9.717	17.17 +/- 23.686	0.026*

Both symptomatic cholelithiasis and dyskinesia were identified to be the most common indicators for surgical intervention in 36 cases (34.6%) of SIRC. Among MIRC cases, symptomatic cholelithiasis was the most common indicator 53 (50.5%) cases, followed by dyskinesia in 25 (23.8%) cases. This suggests that dyskinesia was observed in approximately one-third of SIRC and only one-fourth of MIRC cases. Acute and chronic cholecystitis were relatively minimal indicators for the surgery: acute observed in three (3.0%) cases and chronic in seven (6.7%) cases of SIRC, while acute in six (5.7%) and chronic in four (3.8%) cases of MIRC (Table 3).

**Table 3:** Single-incision robotic cholecystectomy and multi-incision robotic cholecystectomy indication for operation SIRC, single-incision robotic cholecystectomy; MIRC, multi-incision robotic cholecystectomy

Indication	SIRC	MIRC
Dyskinesia	36 (34.6%)	25 (23.8%)
Symptomatic cholelithiasis	36 (34.6%)	53 (50.5%)
Acute cholecystitis	3 (2.9%)	6 (5.7%)
Chronic cholecystitis	7 (6.7%)	4 (3.8%)
Unknown	16 (15.4%)	11(10.5%)
Others (choledocholithiasis, polyp, etc.)	6 (5.8%)	6 (5.7%)

Intraoperatively, 45 (42.9%) cases of MIRC received irrigation usually for bile spillage, while 31 (29.8%) cases of SIRC received irrigation for the same reason. The use of firefly visualization use was similar in both cases: 22 (21%) cases of MIRC and 21 (20.2%) of SIRC. Although uncommon, a gallbladder perforation with leakage of bile was noticed in 15 (14.3%) cases of MIRC and 18 (17.3%) of SIRC. Intraoperative cholangiogram was performed in only seven (6.67%) cases of MIRC and eight (7.70%) of SIRC (Table 4). The LOS parameter comprised outpatients in 75 (71.4%) cases of MIRC and 84 (80.8%) SIRC (Table 5). Using Pearson chi-square analysis, there was a significant difference in the use of irrigation and LOS between SIRC and MIRC (Table 6). All other intraoperative events did not significantly differ between the two approaches as the null is rejected.

**Table 4:** Single-incision robotic cholecystectomy and multi-incision robotic cholecystectomy intraop events SIRC, single-incision robotic cholecystectomy; MIRC, multi-incision robotic cholecystectomy

Intraop Events	SIRC	MIRC
Irrigation	31 (29.8%)	45 (42.9%)
Firefly usage	21 (20.2%)	22 (21.0%)
Perforation	18 (17.3%)	15 (14.3%)
Intraoperative cholangiogram	8 (7.70%)	7 (6.67%)

**Table 5:** Single-incision robotic cholecystectomy and multi-incision robotic cholecystectomy length of stay SIRC, single-incision robotic cholecystectomy; MIRC, multi-incision robotic cholecystectomy

Length of stay	SIRC	MIRC
0 days	89 (80.8%)	75 (71.4%)
1 day	7 (14.4%)	17 (16.2%)
>1 day	8 (7.70%)	13 (12.4%)

**Table 6:** Single-incision robotic cholecystectomy and multi-incision robotic cholecystectomy statistical analysis of intraop events *indicates a significant difference between the two approaches

Pearson Chi-square	P-value
Indication	0.1579
Irrigation	0.0499*
Firefly	0.895
Perforation	0.549
Intraoperative cholangiogram	0.773
Length of stay	0.0379*

No cases reviewed required conversion to open or laparoscopic approaches as these were excluded. None of the patients suffered bile duct or any other injuries to surrounding structures. Intraoperatively, there was one case of bowel enterotomy with trocar placement in the multi-incision approach. There was one report of gallbladder enterotomy with gross spillage of stones with bile. Umbilical hernias were found and repaired in three (2.88%) cases of SIRC.

## Discussion

Robotic surgery is becoming more common, tripling over laparoscopic surgery across all disciplines in surgery from 6.8% in 2008 to 17% in 2012 [6]. SIRCs and MIRCs have shown promising results as the most important cholecystectomies. This is supported by the low complication rate due to the control afforded to the surgeon. When comparing MIRC to SIRC in the first nine children to receive elective robotic cholecystectomy in a tertiary-care institution (by a single surgeon), SIRC had a greater total operative time (SIRC 169 minutes vs MIRC 139 minutes), with the difference between the medians being 30 minutes, and greater total console time (69 minutes SIRC vs 47 minutes MIRC), with the difference being 22 minutes [4]. This is quite contradictory to our data, which may be attributed to the learning curve associated with SIRCs compared to MIRCs as well as the experience from the nine surgeons in this study compared to the single surgeon in the tertiary-care institution. Comparing single- and multi-incision approaches for hysterectomies for endometrial cancer, there was a significance difference in blood loss between the two approaches (50 mL single-incision vs 100 mL multi-incision) and total hospital stay (two days single-incision hysterectomy vs three days multi-incision hysterectomy), while the total operative time has been found to be similar among the two (110 minutes single-incision hysterectomy vs 102.5 minutes multi-incision hysterectomy) [7]. Our results affirm that single-incision has less total hospital stay, likely attributed to the avoidance of vasculature with the single incision residing in midline while multi-incision incisions made away from the midline, the risk being placing through an inferior epigastric or another blood vessel.

Laparoscopic approaches, on the other hand, do not offer much difference in outcomes except the robotic-assisted cholecystectomy has been associated with less biliary injuries due to the higher precision [8]. We encountered no identified biliary injuries in our review of 209 patients over five years. The higher precision of robotic surgery has been known since its introduction, but the full feasibility comes in the narrow anatomical areas of the body where the surgeon has the option to adjust the console to robotic instrument movement ratios. Laparoscopic approach to cholecystectomy was found to be inferior to a robotic-assisted approach requiring more conversion, longer operative time, more perioperative bile spillage, and more postop bile spillage. Although the power of these findings remained of low non-significant value, early suggestion of these variables is important to note and follow in the future [3]. Pain level has been found to be less with the robotic approach when compared to laparoscopic approach [7]. This decrease in pain may be related to the focus of the robotic system to place the center of rotation and movement at the patients’ fascia, reducing the overall stress forces encountered in the operation. In comparing robotic-assisted abdomino-perineal resections (APR) vs laparoscopic approach, the robotic approach allowed for better visualization, given the enhanced three-dimensional visualization and precision of the EndoWrist [1,3,9-10].

Driven by a lack of concrete evidence to the benefits of SIRC and MIRC, we conducted a retrospective chart review comparing the clinical and perioperative outcomes of SIRC and MIRC. Our comparison consisted of 209 patients, 105 MIRC and 104 SIRC cases, retrospectively, in a community hospital. In addition to reviewing the outcomes, we compared operative time, console time, EBL, and any significant intraoperative events and matched these based on patient age and gender. Choice of an SIRC vs MIRC approach was determined by surgeon preference, but SIRC compared to laparoscopic approach is generally favored for patients of lower BMIs [2].

Our report indicates that SIRC has advantages over MIRC including less EBL, supported by previous articles, and less total operative time. We did not find any significant difference in total console time between the two approaches. Intraoperatively, symptomatic cholelithiasis was the overall primary indicator for both SIRC and MIRC cases, with dyskinesia being the second most common indicator, although observed in only 36.4% cases of SIRC and 23.8% of MIRC. Symptomatic cholelithiasis was the primary indicator for 50.5% of MIRC and only 36.4% of SIRC. It is assumed that SIRC patients are not in acute distress and have time for the cosmetically superior SIRC approach, as supported by 36.4% of the SIRC patients having stable dyskinesia vs only 23.8% or MIRC [2]. Intraoperative events did not vary among the two approaches and only irrigation usage was significant only marginally, which is most likely due to surgeon preference. Three SIRC cases had umbilical hernias identified during operation, and no follow-up regarding postop hernias was recorded, but previous articles found a 7.1% occurrence of ventral hernias following SIRC with an average of 27.7 months postop [2]

Our data indicate that the hospital LOS remained lower for SIRC cases, thus reducing the expenditure of SIRC to some extent; however, the instruments and set up involved in SIRC contribute to the high cost when compared to MIRC: perioperative cost SIRC $6158 vs MIRC $4288 USD (*p *< 0.0001) [2]. Pediatric patients undergoing SIRC, when compared against a laparoscopic approach, have half the hospital stay [5]. This cost does not include capital acquisition, team training, maintenance of equipment and repair, and operating room setup time. By saving on the hospital stay, the overall savings outweigh the intraoperative cost. In comparing robotic versus open common bile duct exploration, a net saving of $3000 per case was noted [9]. This is also associated with less hospital-acquired complications with shorter inhospital stay.

## Conclusions

SIRC and MIRC cases are both safe and feasible ways to remove the inflamed/dysfunctional gallbladder. SIRC is associated with less operative time, less blood loss, and shorter hospital stay. Further large-scale studies are needed to make a definitive decision as many of the variables were not significant possibly due to the small single-institution sample size. Although the robotic approach should be individualized based on the surgeon experience/preference, according to our study, SIRCs seemed to have improved outcomes.
